# HSP70 upregulation in nasal mucosa of symptomatic children with allergic rhinitis and potential risk of asthma development

**DOI:** 10.1038/s41598-022-18443-x

**Published:** 2022-08-18

**Authors:** Anna Fagotti, Livia Lucentini, Francesca Simoncelli, Gianandrea La Porta, Leonardo Brustenga, Ilaria Bizzarri, Silvia Trio, Chiara Isidori, Ines Di Rosa, Giuseppe Di Cara

**Affiliations:** 1 Department of Chemistry, Biology and Biotechnology, University of Perugia, Via Elce di Sotto 8, 06123 Perugia, Italy; 2 Pediatric Unit, Department of Medicine and Surgery, University of Perugia, Piazza Lucio Severi 1, 06123 Perugia, Italy

**Keywords:** Cell biology, Molecular biology, Biomarkers, Molecular medicine

## Abstract

Allergic rhinitis and asthma are the most common causes of chronic inflammation of the upper and lower airways in childhood. However, a nasal biomarker that can link to pulmonary inflammation is yet to be found. The present paper aims to investigate the possible role in inflammation of two inducible 70-kDa Heat Shock Proteins (HSP70) members, HSPA1A/B and HSPA6, in nasal mucosa cells of allergic children through their mRNA expression analysis, and their correlation to both spirometric and FeNO values. The relationship between FeNO in lower airways and ∆Cts of HSPA1A/B in nasal mucosa seems to be influenced by clinical symptoms regardless of age, sex, and sensitization patterns. Therefore, HSP70 expression, as well as FeNO levels, could have a predictive capability to identify lower airways inflammation and thus to recognize rhinitic children having a potential risk of asthma development.

## Introduction

Allergic rhinitis (AR) and asthma are the most frequent causes of chronic inflammation of the upper and lower airways in childhood, with a worldwide important impact in terms of quality of life, as they influence both sleep quality and school performance, with direct and indirect economic costs^1,2^. AR is also a significant risk factor for asthma development and an important trigger for asthma exacerbation^3^, as reported in the Allergic Rhinitis and its Impact on Asthma (ARIA) guidelines^4^.

Epidemiological evidence has consistently demonstrated that AR and asthma often occur in the same individuals, with 40–50% of AR patients having concomitant asthma and 70–90% of asthmatics presenting AR as a comorbidity^5–8^. It has been proposed by several authors that AR and asthma may be different manifestations of a global respiratory disease^9^ and a new terminology has been hence introduced, namely "Combined Allergic Rhinitis and Asthma Syndrome (CARAS)"^10,11^. The AR and asthma association is influenced by many interactions between the upper and lower airways^12^. Several studies suggest that AR may increase lung inflammation and reduce asthma control, increasing bronchial reactivity^13,14^. This concept is supported by pathophysiologic evidence, showing that a very similar airway inflammatory pathway is shared by AR and allergic asthma. The same inflammatory cells, including eosinophils, mast cells, and T-helper 2 (Th2) cells, together with pro-inflammatory mediators are present in the nasal and bronchial tissues of patients with AR and asthma, where they are involved in Th2 cell-mediated inflammation and tissue eosinophilia^15–18^.

During the last decades research efforts in clarifying pathophysiologic mechanisms of AR and asthma have changed and improved both diagnostic and therapeutic approaches. A large number of studies have focused on the identification of peculiar biomarkers for these allergic airway pathologies^19,20^.

Although many of them may be useful tools to confirm a diagnosis in case of inconclusive clinical features^21–25^, the identification of a reliable prognostic biomarker, able to select among AR children those at risk of developing asthma, might play a great role in defining and developing appropriate preventive strategies and, if possible, treatments.

In the last decade it has been suggested that fractional exhaled nitric oxide (FeNO), which is a validated and sensitive marker for airway eosinophilic inflammation^23^, could be also used to identify early-onset asthma among children with AR. In fact, it has been demonstrated that in children with higher levels of FeNO, normal spirometric values and no clinical history of preschool wheezing and/or asthma, the prevalence of asthma development was significantly higher than in the control group^26^. Such possible AR endotype with higher risk of asthma development seems to be present in a significant percentage of the paediatric population^27^.

Among inflammation biomarkers, many studies have shown that changes in 70-kDa Heat Shock Proteins (HSP70) expression are involved in the pathogenesis of inflammatory diseases, suggesting its role as a disease marker, therapeutic target, and modulator of inflammation^28,29^. HSP70s are a family of ubiquitarian molecular chaperones with a major role in regulating protein homeostasis by facilitating its folding, assembly and transport. While some HSP70s are constitutively expressed, other members are stress-inducible and play a fundamental cytoprotective role by facilitating protein damage repair in cells exposed to numerous stressful and pathological conditions^29^.

The human HSP70 family consists of 13 members, five of which show a stress-inducible expression, following environmental, pathological or physiological stress^30^. Two closely linked inducible proteins, referred as HspA1Aand HspA1B (collectively named HspA1A/B and coded by *HSPA1A* and *HSPA1B* genes, respectively), constitute the strongest stress-induced proteins playing a critical role in cell survival.

These isoforms are considered interchangeable on the basis of their functions^31^. An additional stress-inducible HSP70 member is the poorly investigated HspA6, encoded by *HSPA6* gene. This isoform results more strictly stress-induced compared to HspA1A/B^30–32^. HSP70 induction has been often associated with an allergic airway inflammatory state of asthma^33^. Specifically, several studies have reported high levels of Hsp70 in the lower airway cells, in serum and sputum demonstrating its involvement in the pathogenesis of asthma and suggesting a role as disease biomarker^34–38^. However, the relationship between upper airway inflammation and expression of HSP70 has not been investigated enough. Few studies reported elevated levels of Hsp70 in nasal secretion and nasal mucosal smears of adult patients affected by AR and chronic rhinosinusitis^39–42^, suggesting that this protein can be used as a disease indicator also for upper airway syndromes.

The main purpose of the research is to investigate the possible role of HSP70 in the nasal mucosa cells of children with AR history through the analysis of mRNA expression of two inducible HSP70 members: HSPA1A/B and HSPA6. In addition, mRNA levels have been examined in relation to both spirometric and FeNO values, in order to define the possible role of HSP70 in nasal mucosa of allergic children as a possible biomarker of lower airways inflammation and asthma evolution.

As HSP70 may be involved in several inflammatory diseases, thus being influenced by several environmental and endogenous stimuli, thestudy includes only patients with this single and specific inflammatory condition in different phases of clinical activity, with no healthy control group, in order to minimize any possible interference due to non-recognized cofactors, that could lead to a higher variability of data results. The choice to include AR patients with different phases of clinical disease was aimed at better defining if higher levels of activity in upper airways disease may increase the possibility to detect an involvement of lower airways, increasing accuracy of this possible screening method. For this reason, among those patients we have also tried to define whether different patterns of allergen exposures, particularly seasonal (pollens) or perennial (house dust mites, HDM), could impact significantly on HSP70 expression and on a possible correlation with lower airways inflammation.

## Results

**Figure 1:**
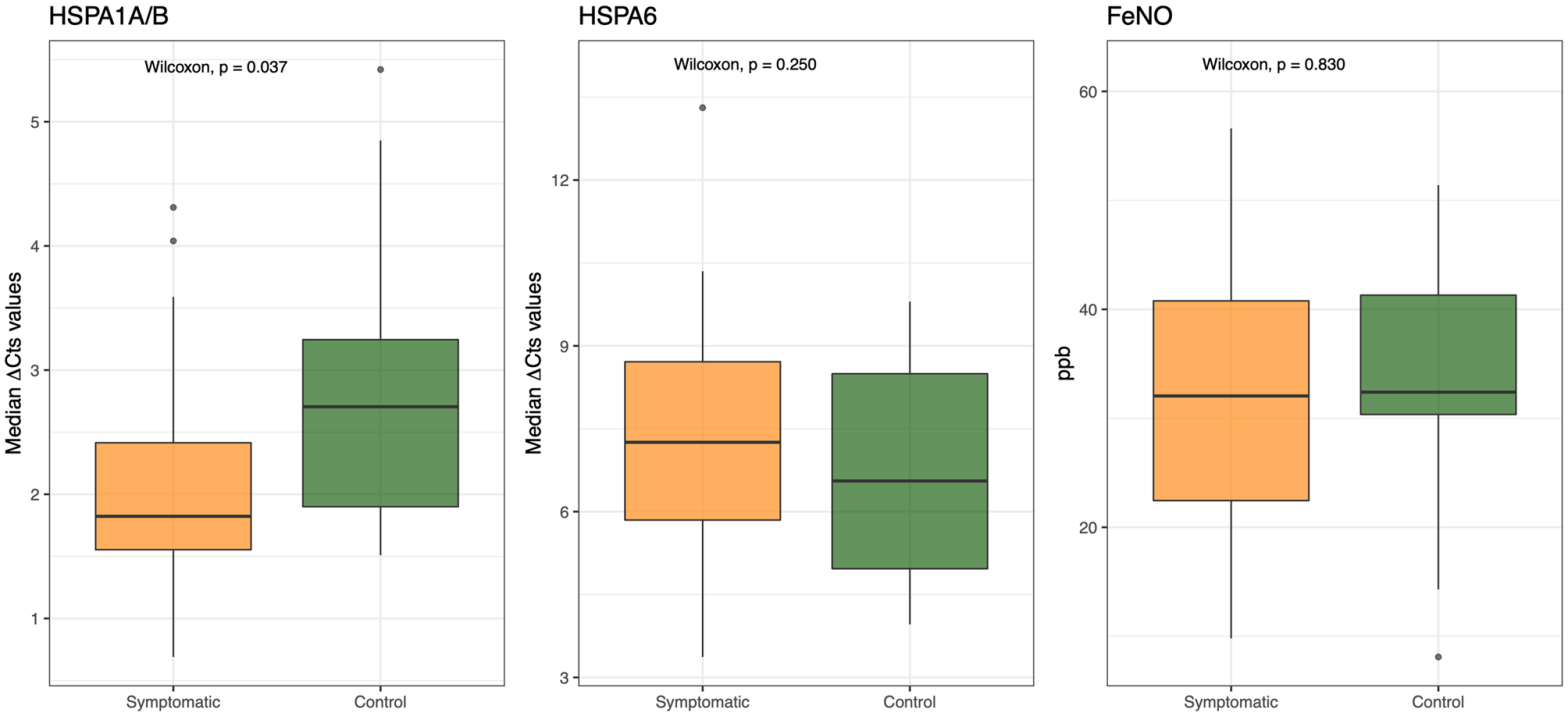
Boxplot of median ∆Ct values for HSPA1A/B and HSPA6 transcripts and ppb values for FeNO in the symptomatic AR patients (orange) and control group (green). Higher ∆Ct values stand for lower mRNA expression.

The HSP70 response has been investigated by evaluating transcript profiles of two different inducible HSP70s coded by *HSPA1A/B* and *HSPA6* genes. Data derived from RT-qPCR have been presented as ΔCT values, with higher ΔCT values standing for lower mRNA expression. As expected, there was no difference in the expression of the two genes between sexes (data not shown), therefore males and females were analyzed together. The results revealed that the two Hsp70 members were differentially expressed: ∆Cts of HSPA1A/B were significantly lower in the nasal cells of AR symptomatic patients compared with control children (Wilcoxon = 167; P < 0.01) (Fig. 1), showing that the HSPA1A/B transcript levels were significantly higher in the nasal cells of AR patients with clinical symptoms compared with asymptomatic children (P < 0.01). Conversely, the HSPA6 transcripts were lightly downregulated (higher ∆Cts) compared to controls, but the differences were not statistically significant (Wilcoxon = 236; P > 0.05) (Fig. 1). At the same time, no statistically significant differences emerged for FeNO between the two groups (Wilcoxon = 248; P > 0.05), suggesting no significant eosinophilic inflammation differences in the lower airways of those patients.

**Figure 2:**
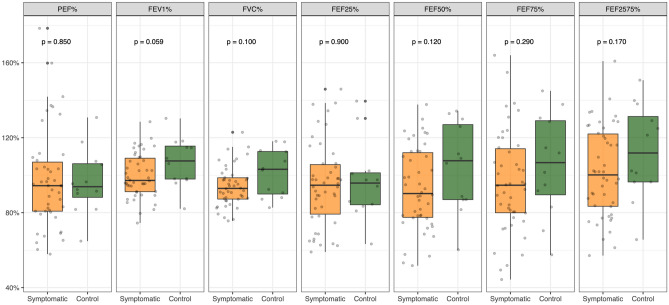
Spirometric patterns in the AR symptomatic subjects (orange) and control group (green).

In order to verify the presence of differences between the two groups according to the disease state activity, and to exclude underlying and unrecognized asthma, a spirometry test was performed. As expected, the spirometric patterns showed no significant differences between the two groups, for none of the considered variables (Fig. 2).

**Figure 3:**
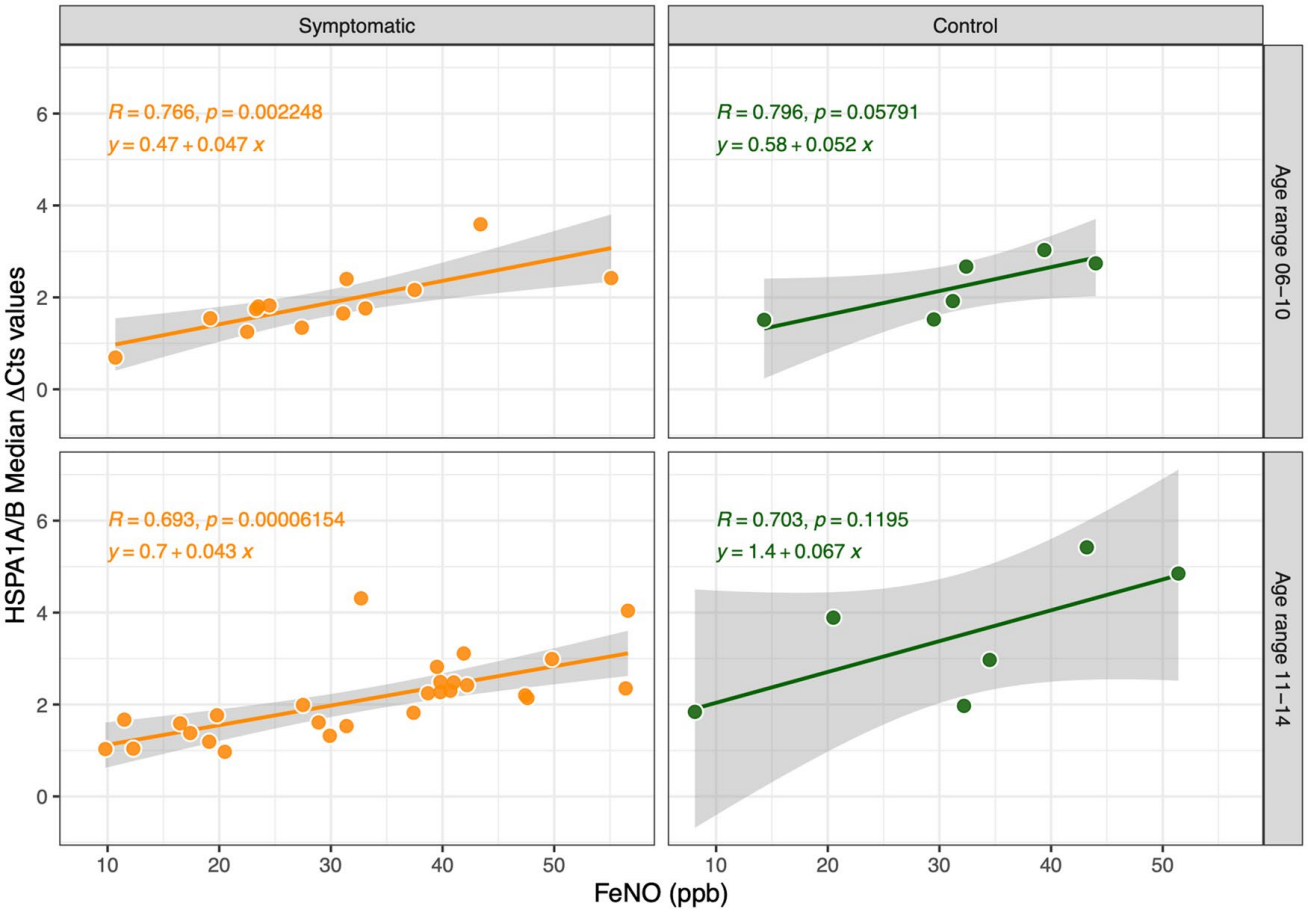
Regression and correlation between FeNO concentration and ∆Cts of HSPA1A/B in AR subjects and control group, separated into two age ranges.

Spearman's rank test revealed significant and positive correlations between the variables. In particular, ∆Cts of HSPA1A/B and FeNO were strongly and significantly correlated in AR symptomatic subjects for both age ranges (R_6-10_ = 0.766, P < 0.01; R_11-14_ = 0.693, P < 0.001); similarly, FeNO was positively correlated with ∆Cts of HSPA1A/B in control cases, even though R values (R_{_6–10_} = 0.796, P > 0.05; R_{_11–14_} = 0.703, P > 0.05) began to lose significance (Fig. 3).

**Figure 4:**
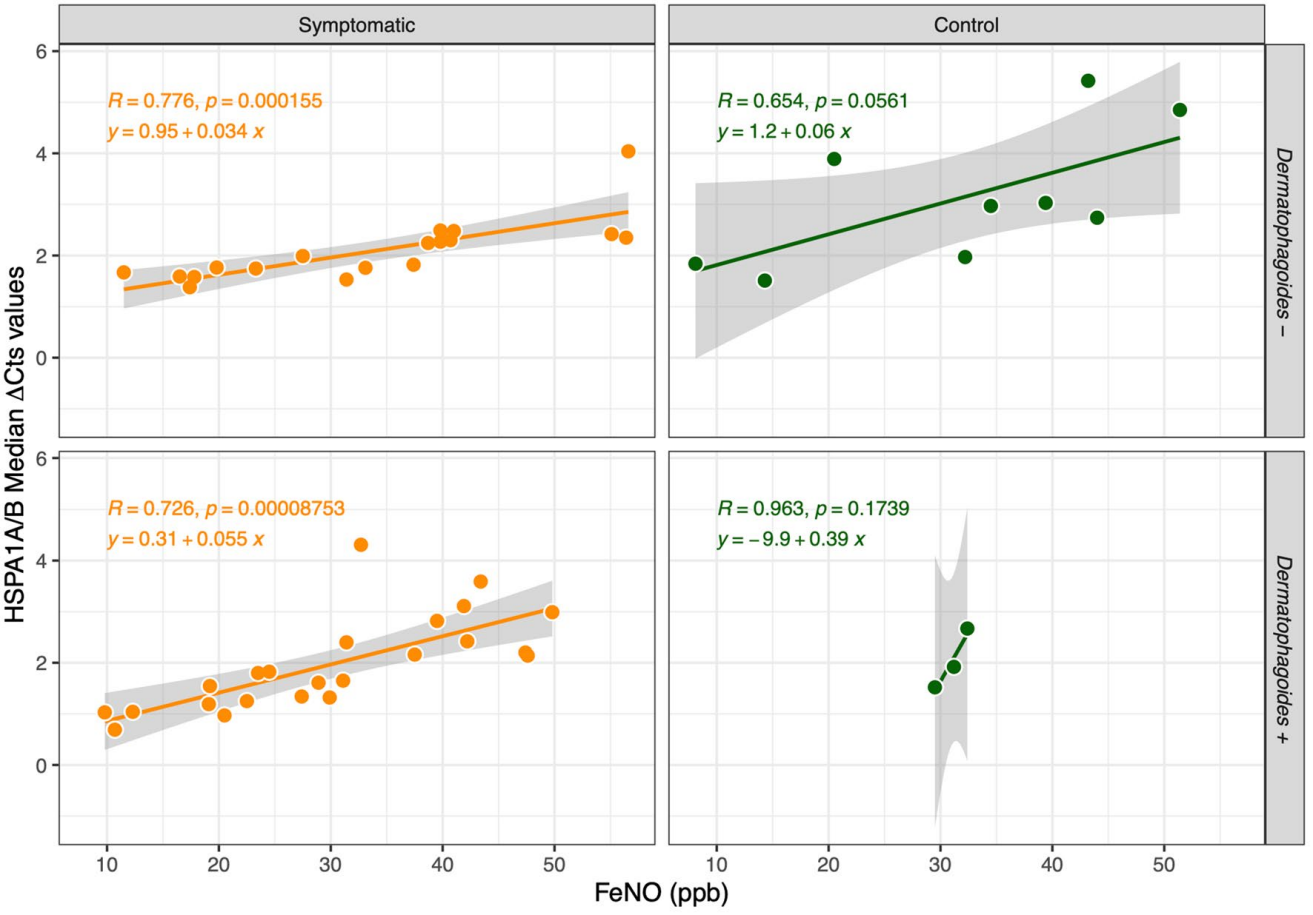
Regression and correlation between FeNO concentration and ∆Cts of HSPA1A/B in AR symptomatic subjects and control group, separated by *Dermatophagoides* allergens sensitization.

The role of a perennial exposure to allergens, such as HDM, with a persisting inflammatory stimulus as a possible cofactor of transmission from upper to lower airways, was also considered. A similar trend was observed separating data according to the presence/absence of sensitization to *Dermatophagoides* allergens, with significant and positive correlations between FeNO concentration and ∆Cts of HSPA1A/B (R values in plots), independently from sensitization pattern (Fig. 4).

**Figure 5:**
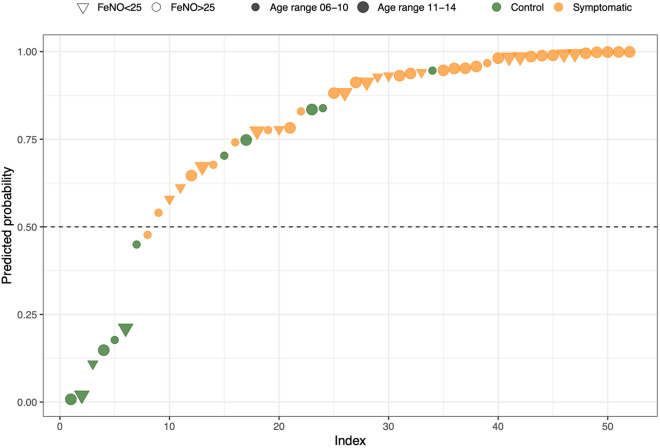
Ranking of AR symptomatic (orange) and control (green) patients, based on likelihood of showing symptoms, differentiated by FeNO measurement levels (triangle FeNO < 25, circle FeNO ≥ 25) and age range (smaller shape 6–10, bigger shape 11–14).

To assess how much the independent variables HSPA1A/B, FeNO, FVC%, FEV1%, sensitization to *Dermatophagoides* allergens, and age impacted on the allergic status, a logistic regression model was implemented. This model had adequate predictive accuracy (McFadden pseudo-R^2^ = 0.41), with the included variables showing vif values ranging from 1.5 to 3 (Table 1). The overall classification rate was 88%, with a 97% correct classification of the symptomatic cases (Fig. 5). We also used the fitted logistic regression model to predict the probability that, in a given individual, asthma and asthma evolution signs occurred, based on the concentration of FeNO and the HSPA1A/B expression. A unit increase in FeNO led the odds of showing symptoms to increase by a factor of 1.12 and a unit increase in ∆Ct of HSPA1A/B to decrease it by a factor of 0.13. In other words, the odds ratio of asthma symptoms increased by a factor 7.7 for a unit decrease in ∆Ct of HSPA1A/B.

**Table 1:** Logistic regression model coefficients.

Term	Estimate	Std.error	Statistic	P value
Intercept	6.20	4.09	1.52	0.129
HSPA1A/B	−2.04	0.79	−2.57	0.010
FeNO	0.12	0.06	2.07	0.038
FVC%	−0.04	0.06	−0.65	0.516
FEV1%	−0.02	0.06	−0.38	0.702
*Dermatophagoides* sensitization	2.32	1.08	2.14	0.032
Age group 11–14	2.96	1.21	2.46	0.014

An even stronger association with the symptoms was observed for the *Dermatophagoides* allergens sensitization with an odds ratio of 10.2, and with an older age with an odds ratio of 19, whereas FVC% and FEV1% showed odds ratios close to 1: 0.96 and 0.98 respectively.

## Discussion

Growing evidence indicates that there is a close relationship between upregulation of HSP70 expression and the pathogenesis of allergic diseases, suggesting that this chaperone could be employed as a significant disease biomarker^33,42^. However, to date, only a few studies have considered HSP70 expression in patients with allergic rhinitis and, to the best of our knowledge, no data are present in childhood. The current study is the first report that analyses the HSP70-mediated response in nasal mucosa cells of children with AR by using a simple and well tolerated scraping technique as the cell collection procedure. Due to its minimal invasiveness and good reproducibility, this sampling method can be easily performed in paediatric patients and applied to study AR diagnostic markers in the clinical practice.

The HSP70 response has been investigated by evaluating transcript profiles of two different inducible HSP70s coded by *HSPA1A/B* and *HSPA6* genes. The results revealed that the two members were differentially expressed: HSPA1A/B levels were significantly increased in the nasal cells of symptomatic patients compared to asymptomatic children. Conversely, the HSPA6 transcripts were downregulated compared to controls but the differences were not significant.

Although data are lacking on the contribution of the individual HSP70 inducible isoforms in airway allergic inflammation, some studies in human stressed cells show that Hsp70-1 and Hsp70-6 isoforms, encoded respectively by *HSPA1A/B* and *HSPA6* genes, have different activation and regulation pattern^30,32,43^. *HSPA1A/B* results to be the major heat-inducible gene in humans responding to numerous cytotoxic stimuli, whereas *HSPA6* is characterized by tight regulation, showing to respond to extreme stress conditions and to be induced to complement the levels of *HSPA1A/B* in the cytoprotective function. The difference in the transcriptional activity of *HSPA1A/B* and *HSPA6* genes in nasal mucosa cells of symptomatic children may reflect their distinct functional roles during airway inflammation.

The presence of a HSP70 response in children agrees with a few results observed in adult AR patients. Specifically, the study of Chalastras et al*.*^39^ demonstrated a significantly positive immunoreactivity for Hsp70 in nasal mucosal smears of adult seasonal AR patients compared with control subjects. Moreover, elevated levels of Hsp70 protein were observed in nasal secretions, and its association with the severity of inflammation of upper airway mucosa was demonstrated both in allergic rhinitis^40^ and in chronic rhinosinusitis of adult patients^41,42^. Similarly to these findings, many investigations reported an HSP70 expression increase in airway cells, secretions, and in the peripheral circulation of asthmatic patients^34–36,38^, suggesting that this chaperone is important in the pathogenesis of allergic asthma. Many studies concerning inflammatory diseases have evidenced that HSP70 may play a dual immunomodulatory function both as a pro-inflammatory and anti-inflammatory mediator, also depending on the intracellular or extracellular location of the protein. However, the function that HSP70 exerts in allergic airway inflammations is still largely unknown. Interestingly, a recent work by Yombo et al*.*^44^ has reported the critical role of HSP70 as a positive modulator of allergic airway inflammation. Specifically, by using a mouse model of allergic airway inflammation induced by *Schistosoma mansoni* soluble egg antigen (SEA), the study has demonstrated that Hsp70 is involved in the amplification of Th2 cell activation and in Th2 cytokines production and that the lack of inducible Hsp70s in HSP70 double knockout (Hsp70.1/0.3 mice) results in a significant reduction in eosinophil level and Th2 cytokine production compared with the WT mice. Besides these pro-inflammatory effects, a few studies showed that Hsp70 can also act as a negative mediator during allergic airway inflammatory processes^33^. In the present research, the different pattern of both HSPA1A/B and HSPA6 transcripts in children with a defined allergic inflammation, but with different stages of activity, suggests that HSP70 could be more related to the rapid response to inflammatory stimuli and allergen exposure. In particular, it is possible to assume that the inducible isoform encoded by *HSPA1A/B* gene may play a key role in the inflammatory response mediated by the Th2 pathway. A wider detection of HSP70 variability in different children with different clinical stages of disease and duration of symptoms will define a possible role also in late-phase allergic response and its impact on airways remodelling.

The correlations evidenced between FeNO concentration in lower airways and ∆Cts of *HSPA1A/B* transcript in nasal mucosa seem to be independent of age, sex, and sensitization patterns, being influenced only by clinical symptoms. In our population, the presence of a sensitization to perennial allergens, such as HDM, was not related to significant variations in ∆Cts of HSPA1A/B and to lower airways eosinophilis inflammation (no significant increase of FeNO) when compared to HDM negative AR children. Although these data should be confirmed in a wider population, this result could suggest that HSP70 detection has a direct correlation with FeNO, and is not influenced by the duration of the upper airways inflammatory stimulus. If these data will be confirmed, we could consider HSP70 to be a positive modulator of the upper and lower allergic inflammation which could be used as a biomarker of asthma development risk. As this lack of correlation with SPT sensitization, and thus to the duration of inflammation, supports the hypothesis of a direct link between *HSPA1A/B* expression and lower airways inflammatory signals, these data would suggest a potential usefulness of HSP70 also in asthma of non-allergic origin. A better picture of such hypothesis should however be defined by further studies, comparing nasal *HSPA1A/B* with FeNO also in asthmatic children and in children with other causes of chronic bronchial inflammation.

Several studies support the evidence that an increase in FeNO levels, reflecting lower airways eosinophilic inflammation, may precede both spirometric alterations and asthmatic symptoms. If our results could be confirmed in a wider population, the use of nasal HSP70 detection could therefore play a similar role.

The identification of a nasal biomarker able to define a risk of asthma development in children with AR only, could be used to select different follow-up pathways of children with upper airways symptoms and allergic sensitization.

Moreover, if this result could be confirmed in preschool children, such strategy could be used also in children under 5 years of age, who are unable to perform correctly both spirometry and FeNO measurement, to better define their risk of asthma development.

Even though a larger number of patients, and the knowledge of specific triggers, should be considered to evaluate the consistency of our data, including patients from different geographic areas and more representative preschool age, the present paper represents the first and important observation of the role and interaction of molecular HSP70-mediated processes with other diagnostic and monitoring tools in airway inflammation.

## Methods

### Patients

The study has been performed in accordance with the principles stated in the Declaration of Helsinki. Prior to starting the study, ethical approval of our protocol has been obtained from our ethics committee ‘Comitato Etico Aziende Sanitarie della Regione Umbria (CEAS Umbria)’, which confirmed that the study met national and international guidelines for research on humans.

**Figure 6:**
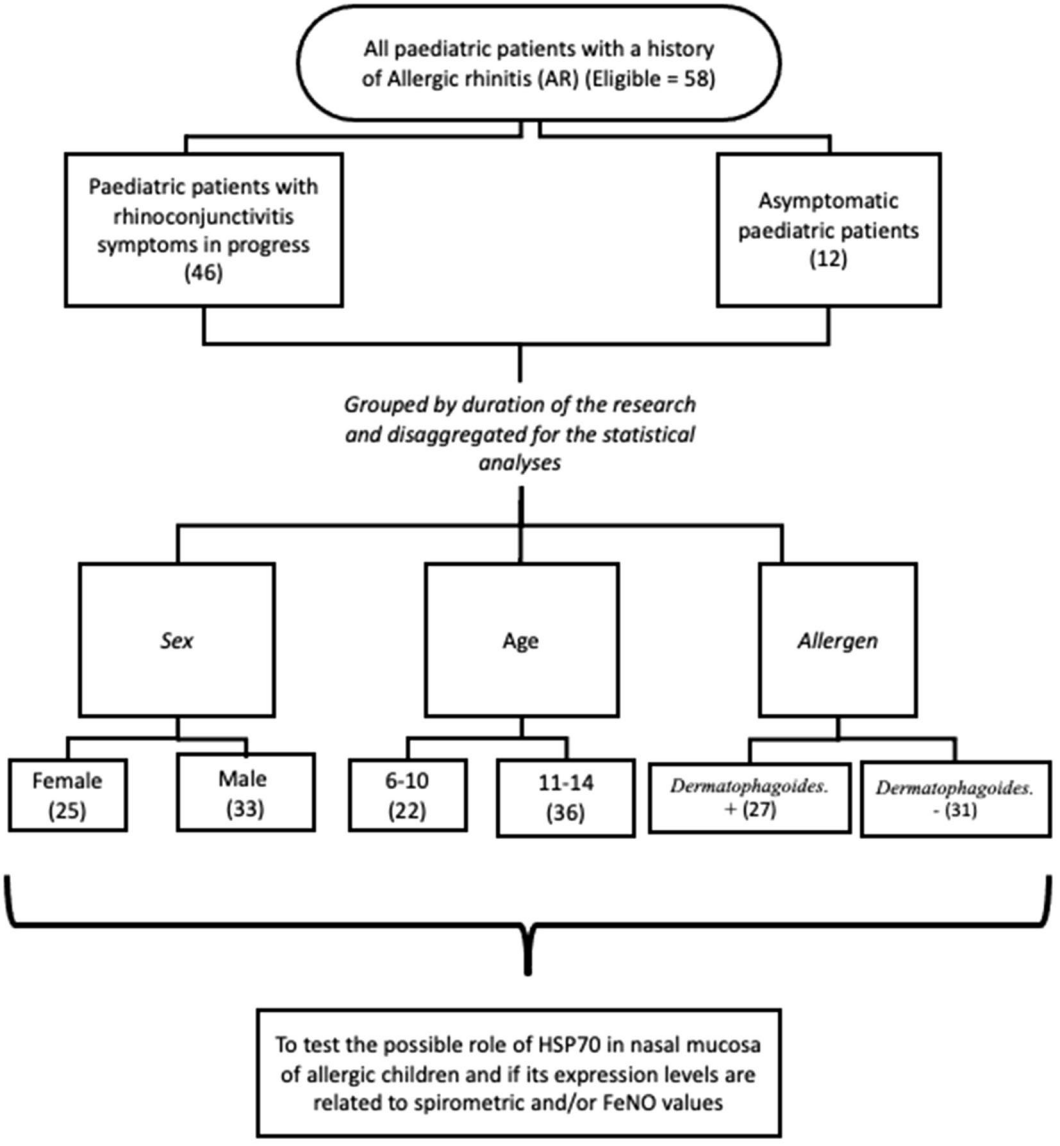
Flow chart reporting patient’s enrollment in the study.

58 paediatric patients (33 males, 25 females, mean age 11.03 ± 2.41 years, age range 6–14 years) suffering from AR, recruited among patients attending the paediatric immuno-allergology service, were included in our study (Fig. 6).

The diagnosis of allergic rhinitis was performed on the basis of the presence of typical clinical symptoms, including itching, nasal congestion, sneezing, and rhinorrhea, which occurred during allergen exposure, according to specific positivity in skin prick tests and/or specific serum IgE^45^. Inclusion criteria were as follows: age range between 6 and 14 years, documented and validated diagnosis of AR with at least one sensitization to inhaled allergens at skin prick test (SPT), and written informed consent signed by parents. Exclusion criteria were as follows: absence of sensitization, history of asthma symptoms, impaired lung function, previous or current specific immunotherapy, and chronic diseases.

Following the verification of his/her personal history concerning upper airways symptoms, each patient was evaluated by performing SPT that tested a positivity for allergen inhalation (pollen from plants or trees, dust mites, animals, moulds). Furthermore, during the screening visit, the patients underwent a complete objective examination to evaluate their overall situation and verifying the inclusion and exclusion criteria.

On the basis of the first visit, paediatric patients were divided into two groups: patients with concomitant rhinoconjunctivitis symptoms were included in the symptomatic group while patients with previous rhinoconjunctivitis history and diagnosis but with no active symptoms during our evaluation, possibly due to lower exposure and/or lower personal reactivity, constituted the control group.

### Skin prick test

SPT was performed as stated by the European Academy of Allergy and Clinical Immunology. The panel consisted of HDM (*Dermatophagoides farinae* and *D. pteronyssinus*), cat, dog, grasses mix, mugwort, ragweed, *Parietaria officinalis*, birch, hazel, olive tree, cypress, *Alternaria tenuis*, *Cladosporium*, *Aspergillus* mix (Stallergenes, Milan, Italy), a positive control (histamine 10 mg/mL) and negative control (physiological solution). The obtained wheals were evaluated as the mean of larger and smaller diameters, considering as a threshold positivity an average of 5 mm.

### Spirometry

Spirometry was performed with a Microloop Micro Medical computer-assisted spirometer (Micro Medical, Rochester, England). The subjects were asked to perform three times a maximum expiratory effort immediately following a maximum inspiration, with a rapid start, and the best forced expiratory volume in 1 s (FEV1) was registered. The test was performed and interpreted as stated by the European Respiratory Society (ERS) guidelines^46^.

### FeNO measurement

FeNO was measured using a chemo-luminescence analyser (NIOX MINO, Aerocrine, Solna, Sweden) according to the international ATS/ERS guidelines^47^. The measurement was performed instructing patients to inhale through the device from the functional residual capacity to the total lung capacity, followed by exhaling into the device for 10 s with a flow rate of 50 ml/s. To obtain a valid result, each patient performed the measurement for three consecutive attempts. FeNO measurement was performed before spirometry. Values below 25 parts per billion (ppb) were considered normal^48^.

### Sampling through nasal mucosa scraping and RNA extraction

Considering both the age of the subjects involved in the study and the necessity to develop a monitoring protocol for paediatric subjects to be evaluated for FeNO and spirometry, it was considered important to proceed with a non-invasive approach. For this reason, scraping of the nasal mucosa was chosen as a non-invasive method to collect nasal samples from children, without local anaesthesia. To remove the mucus, the nasal mucosa was washed with physiological saline solution, and then, by using a nasal speculum, nasal mucosa cells were harvested by gently scraping (two or three times) the medial surface of inferior turbinate with plastic single-use curette (Nasal Scraping, E.P. Medica, Ravenna, Italy). Samples obtained from both nostrils of each patient were pooled by submerging the curettes in Lysis Buffer plus β-mercaptoethanol and then frozen at −80 °C until RNA extraction.

Total RNA was extracted by using the PureLink RNA Mini Kit (Life Technologies, Carlsbad, CA USA) and treated with PureLink DNase (Life Technologies, Carlsbad, CA USA) to remove any traces of remaining genomic DNA. RNA concentration and quality were assessed by the ratio of 260/280 nm absorbance and electrophoresis on 1% agarose gel under denaturing conditions. The purified RNA was stored at − 80 °C until conversion into cDNA.

### cDNA synthesis and analysis of Hsp70 mRNA expression by real-time PCR (qRT-PCR)

First-strand cDNA synthesis was performed by using ImProm-II Reverse Transcription System kit (Promega, Madison, WI, USA) in a total volume of 20 µl in the presence of 0.5 μg of random hexamer primers (Life Technologies, Carlsbad, CA, USA) following the manufacturer's protocol. Reverse transcription was performed at 37 °C for 60 min. Retrotranscription success was tested amplifying the samples with Master Mix Go Taq Hot Start 2x (Promega, Madison, WI, USA); the reaction was carried out in a total volume of 25 µl, including 12.5 of Master Mix, 1 µl of each 10 µM primer, 9 µl of sterilized distilled water and 1.5 µl of cDNA. The amplification program included 2 min at 95 °C followed by 40 cycles of 30 s at 95 °C, 30 s at 60 °C and 30 s at 72 °C, followed by a final extension of 10 min at 72 °C, and it was the same for all primers tested in the project. Amplicons were checked by electrophoretic run with TBE 1× (Promega, Madison, WI, USA) on a 2% agarose gel; to allow UV reading, the gel was treated with SafeView (NBS biologicals, NEB, Huntingdon, UK).

mRNA quantification of inducible HSP70 members, encoded by *HSPA1A/B* and *HSPA6* genes, was then performed by quantitative Real-Time RT-PCR (qRT-PCR) using the gene-specific primer set designed by Boyko et al*.*^49^. To validate the data, preliminary assays were also performed with gene primer set designed for this study by using Primer3 Plus software.

The real-time PCR assays were carried out in a LightCycler^®^ 96 Instrument (Roche Applied Science, Basel, CH) using the FastStart Essential DNA Green Master (Roche Applied Science, Basel, CH). β-actin gene was used as an internal reference gene for normalization purposes. The amplifications were performed in duplicate in a 20 μl reaction volume containing 1.5 μl of undiluted random-primed cDNA template, 1× FastStart Essential DNA Green Master mix, and 0.25 μM of each primer according to the manufacturer's instructions. The PCR program consisted of incubation at 95 °C for 10 min, followed by 40 cycles of a classical three-step amplification (denaturation, 95 °C for 10 s; annealing, 60 °C for 20 s and extension, 72 °C for 10 s) and a final melting phase structured in three steps (10 s at 95 °C, 30 s at 60 °C, 1 s a 97 °C). At the end of each PCR amplification, a melting curve analysis was performed to verify the specificity and define the Tm of each amplicon. Negative control reactions (no-template control) were included in each primer assay to ensure the absence of contamination. PCR efficiency was checked to be at near 100% for each pair of primers. After each run, real-time PCR data were analyzed with LightCycler^®^ 96 Instrument Software 1.0. The relative expression levels of the target genes were determined by the Delta Ct (ΔCt) method where ΔCt was obtained by subtracting the reference gene Ct from the target gene Ct^50^. The sequencing of a sample for each product peak was also carried out and checked for consistency; sequencing was performed following the amplification reported above, purifying the amplicons through an EXO plus SAP protocol (Thermo Fisher Scientific, MA, USA) and then outsourcing to a sequencing service (Eurofins Genomics, Ebersberg, D).

### Statistical analysis

Statistical analyses and calculations were conducted with the R statistical framework (cran.r-project.org), within the RStudio Development Environment (rstudio.com).

The Wilcoxon test was applied for comparisons of *HSPA1A/B* and *HSPA6* gene transcript profiles and FeNO measurements in symptomatic and control patients and to investigate the differences between spirometric patterns in the two groups of children.

The Spearman's rank correlation test was used for correlation analyses between FeNO concentration and ∆Cts of HSPA1A/B in AR symptomatic subjects and the control group. In addition, in order to verify the possible impact of puberty, both groups of patients were evaluated according to age, by distinguishing two age groups: under and over 11 years.

Differences were considered statistically significant at P < 0.05.

A logistic regression model was performed to examine the relationship between patient status (symptomatic or control) and the following dependent variables: HSPA1A/B, FeNO, FVC%, FEV1%, sensitization to *Dermatophagoides* allergens, and age group. Multicollinearity among predictors was checked using the vif function of the R package 'car’.

## Data Availability

The datasets of this study are available from the corresponding author on reasonable request.
